# Genotype-phenotype correlations of adult-onset *PLA2G6*-associated Neurodegeneration: case series and literature review

**DOI:** 10.1186/s12883-020-01684-6

**Published:** 2020-03-17

**Authors:** Yung-Tsai Chu, Han-Yi Lin, Pei-Lung Chen, Chin-Hsien Lin

**Affiliations:** 1 Department of Neurology, National Taiwan University Hospital, No. 7, Chung-Shan South Road, Taipei, 100 Taiwan; 2 Graduate Institute of Medical Genomics and Proteomics, National Taiwan University College of Medicine, Taipei, Taiwan; 3 Department of Medical Genetics, National Taiwan University Hospital, Taipei, Taiwan

**Keywords:** PLA2G6, Dystonia-parkinsonism, Early-onset parkinsonism, Hereditary spastic paraparesis, Ataxia, PLA2G6-associated neurodegeneration

## Abstract

**Background:**

Phospholipase A2 group VI (*PLA2G6*) mutations associated with neurodegeneration (PLAN) manifest as heterogeneous neurodegenerative disorders with variable ages of onset. The genotype-phenotype correlation is not well-established. We aim to describe three adult patients with PLAN and combined these data with results from previous studies to elucidate adult-onset *PLA2G6* phenotype-genotype correlations.

**Case presentations:**

The first index patient presented with dystonia-parkinsonism starting at age 31 years, accompanied by major depression and cognitive decline. Genetic analysis using targeted next generation sequencing (NGS) panel, Sanger sequencing, and segregation analyses revealed a compound heterozygous mutation, c.991G > T (p.D331Y)/c.1077G > A (M358IfsX), in *PLA2G6*. The other two patients had levodopa-responsive, early-onset parkinsonism, starting in their late twenties. Both patients had homozygous c.991G > T (p.D331Y) mutations in *PLA2G6.* Patient characteristics of our reported 3 cases were compared to those of 32 previously described (2008 to 2019) patients with adult-onset PLAN. Among the combined cohort of 35 patients with adult-onset PLAN, 14 had dystonia-parkinsonism, 17 had early-onset Parkinson’s disease, 3 had hereditary spastic paraparesis, and one had ataxia. The c.991G > T (p. D331Y) mutation was almost exclusively found in Chinese patients, suggesting a common founder effect. All patients with homozygous p.D331Y mutations had levodopa-responsive, early-onset PD (100%); while other mutations mostly led to dystonia-parkinsonism, ataxia, spasticity, and combine psychiatric comorbidities.

**Conclusions:**

We showed that adult-onset PLAN could present as purely parkinsonism features, without brain iron accumulation, particularly patients with homozygous p.D331Y mutations. Compound heterozygous mutations, including heterozygous p.D331Y, produced heterogeneous phenotypes, without obvious levodopa responsiveness.

## Background

*PLA2G6*-associated neurodegeneration (PLAN) is a heterogeneous group of neurodegenerative disorders that result from mutations in the phospholipase A2 group VI gene (*PLA2G6*) [1, 2]. The *PLA2G6* gene encodes a group of VIA calcium-independent phospholipase A2 proteins. Phospholipase A2 is an enzyme involved in phospholipid metabolism, and it is essential for maintaining cell membrane integrity [1, 2]. PLAN can be classified into four subtypes, based on onset age, including: infantile neuroaxonal dystrophy (INAD), atypical neuroaxonal dystrophy (ANAD), dystonia-parkinsonism, and autosomal recessive early-onset parkinsonism (known as PARK14) [3]. INAD and ANAD onsets occur in childhood. Brain magnetic resonance image (MRI) findings have revealed that most patients have cerebellar cortical atrophy and iron deposition in the globus pallidus and substantia nigra. These findings are known as neurodegeneration with brain iron accumulation, type II (NBIA II) [4]. In contrast to childhood-onset PLAN, adult-onset PLAN is associated with widely variable clinical manifestations, and the genotype-phenotype correlation has not been well established. Here, we describe three patients with adult-onset PLAN. We compared the clinical features, treatment responses, and radiological findings of these patients to those reported for 43 patients described in previous studies to elucidate the phenotype-genotype correlations in adult-onset PLAN.

## Case presentation

**Fig. 1:**
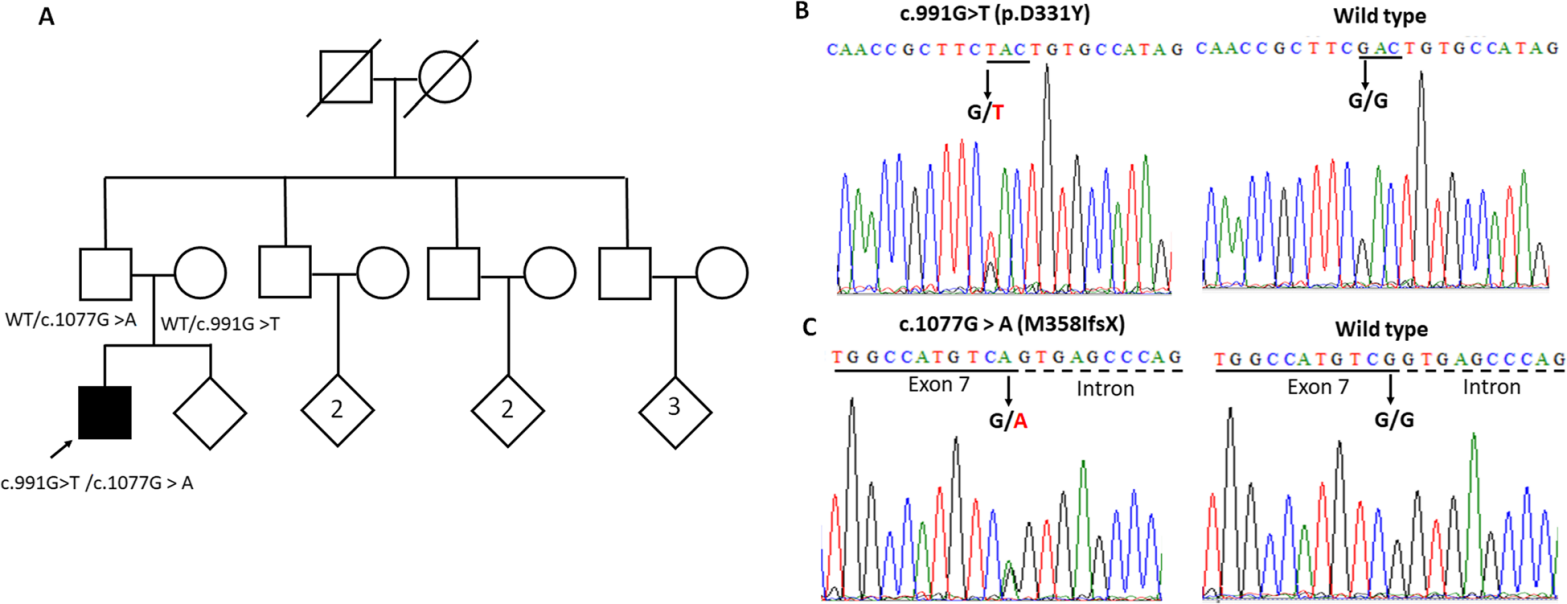
Family pedigree and genetic analysis of an index family. (**a**) Pedigree of an index family with compound heterozygous c.991G > T (p.Asp331Tyr) and c.1077G > A (M358IfsX) mutations in *PLA2G6*. WT, wild type. Open symbol: unaffected; filled symbol: affected; symbol with a diagonal line: deceased; diamond: total number of children, unknown sex; arrow: proband. (**b**) Sanger sequencing traces confirm the heterozygous *PLA2G6* c.991G > T (p.Asp331Tyr) missense mutation. (**c**) Sanger sequencing traces confirm the heterozygous *PLA2G6* c.1077G > A (M358IfsX) frame-shift mutation

Patient 1 was a 36-year-old man, with normal birth and developmental milestones. He had a history of depression for 18 years but had not used anti-depressant or neuroleptic agents consistently. Several times, suicide was attempted. At age of 31 years, motor symptoms gradually developed. He had progressive onset of spastic dysarthria and hypophonia, predominantly left-sided bradykinesia, and dystonia in the left hand ensued. Three years after the onset of motor symptoms, he was confined to a wheelchair with anarthria, and he was totally dependent on assistance from others in daily living activities. In addition, he experienced several episodes of oculogyric crisis. He did not have a relevant family history (Fig. 1a).

**Fig. 2:**
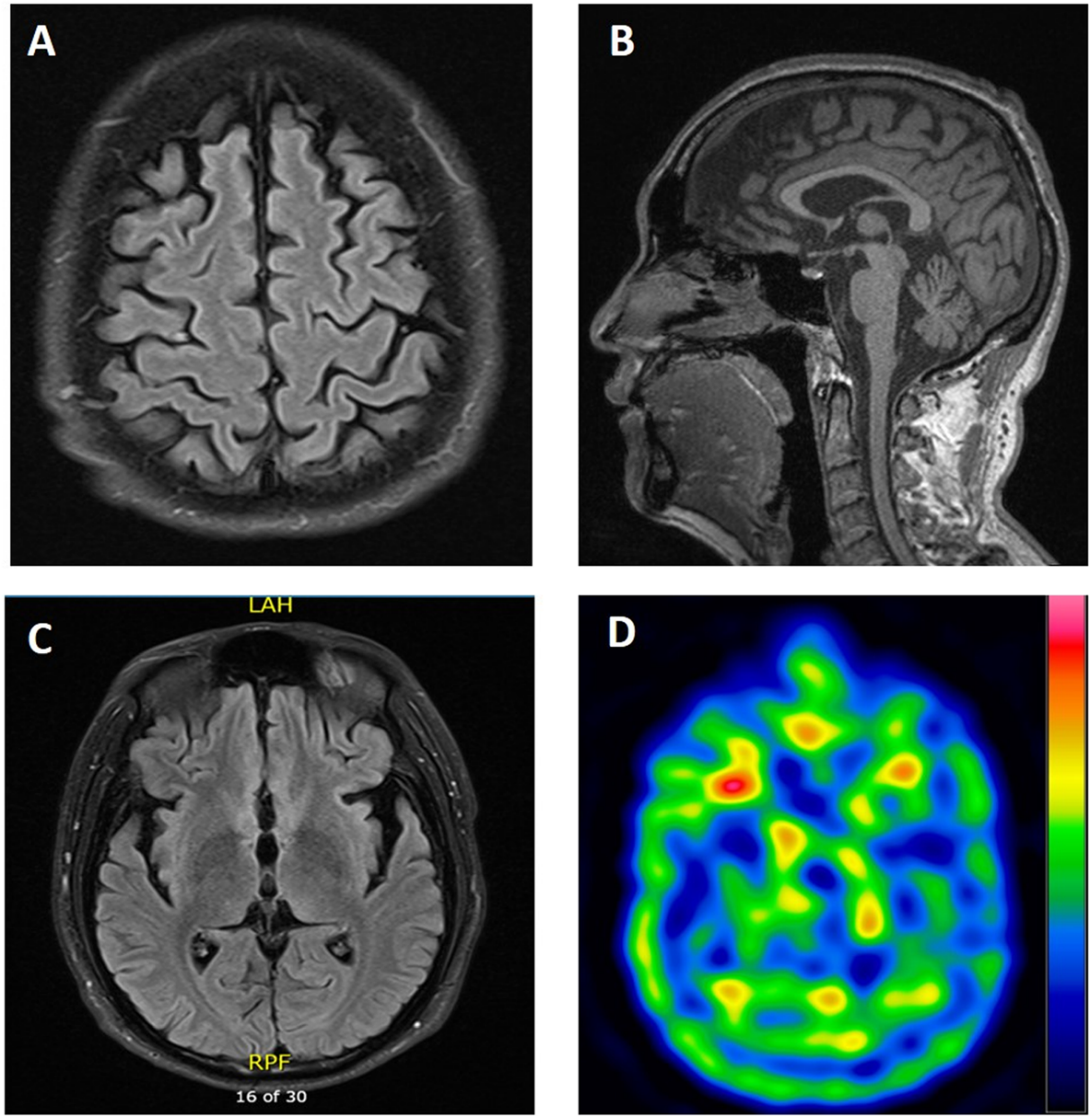
Brain imaging findings in the first index patient. (**a, b, c**) Brain MRIs with T2 Fluid Attenuated inversion recovery (FLAIR) sequence show (**a**) diffuse atrophy in the cortex and cerebellum and (**b**) cerebellar atrophy, but (**c**) no abnormal signs of iron accumulation in the globus pallidus. (**d**) A Tc-99 m TRODAT scan reveals reduced dopamine transporter activity in the bilateral basal ganglia

He was then brought to our movement disorder clinic for evaluation at the age of 36 years. The neurological examination revealed oromandibular, truncal, and limb dystonia and generalized rigidity and bradykinesia; the part III motor score of the Unified Parkinson’s Disease Rating Scale (UPDRS) was 60 out of 108. He scored 21 out of 30 on the Mini-Mental State Examination (MMSE). There were no Kayser-Fleischer rings during the physical examination. A brain MRI showed diffuse cortical and cerebellar atrophy, with no abnormal iron deposits in the basal ganglia (Fig. 2a-c). A Tc-99 m TRODAT single-photon emission computed tomography scan revealed markedly reduced dopamine transporter activity in the bilateral basal ganglia (Fig. 2d). ^18^F-labeled fluoro-deoxyglucose positron emission tomography (FDG-PET) scan demonstrated hypometabolism in the bilateral parieto-occipital lobes. The patient was treated with levodopa (400 mg per day) and benserazide (50 mg three times per day), a rotigotine transdermal patch (8 mg per day), and clonazepam (0.5 mg). These medications improved motor function to the extent that he could walk with assistance, and the UPDRS part III motor score was 40 out of 108 after treatment.

The results of laboratory tests were within normal limits, including normal plasma ceruloplasmin levels and 24-h urine copper excretion. We then performed comprehensive genetic analyses for the index patient with a capture-based next generational sequencing targeted gene panel that covered more than 40 genes related to Parkinson’s disease (PD) and neurodegenerative disorders using similar methods described previously [5]. Mutations were verified with Sanger sequencing and dye-terminator chemistry in an Automated Sequencer Genetic Analyzer (model 3100; Applied Biosystems, Foster City, CA, USA). We identified the pathogenic missense variant, c.991G > T (p.D331Y), and a c.1077G > A (M358IfsX) frame-shift mutation in *PLA2G6* (Fig. 1b,c). Direct Sanger sequencing verified the c.991G > T (p.D331Y) missense mutation in both the proband and the asymptomatic mother (Fig. 2a,b). The c.1077G > A (M358IfsX) frame-shift mutation in *PLA2G6* was confirmed with Sanger sequencing in both the proband and the asymptomatic father (Fig. 2a,c). We then confirmed that the proband had compound heterozygosity, with the mutations in a *trans* configuration.

Patient 2 was a 48-year-old woman presented with progressive shuffling gait and decreased right arm swing since age of 30 years. Right-sided rigidity and bradykinesia were noted later. PD was diagnosed, and she responded well to levodopa. She had prominent psychiatric symptoms, including anxiety, depression and paranoid delusions, which symptoms occurred soon after the onset of right-side limbs slowness. Brain MRIs did not show structural lesions. A Tc-99 m TRODAT SPECT study showed reduced dopamine transporter activity in bilateral basal ganglia, with a more severe reduction on the left side. Her parents were cousins and had a consanguineous marriage. A genetic analysis with a targeted NGS panel, covering 40 genes associated with parkinsonism (supplementary Table 1), identified a homozygous c.991G > T (p.D331Y) mutation in *PLA2G6* [5].

Patient 3 was a 34-year-old woman that started to complain of right-leg clumsiness at the age of 26 years. Right-arm clumsiness and gait disturbances followed 2 years later. Levodopa and dopamine agonists were given, with good motor responses; however, motor fluctuations and levodopa-induced dyskinesia developed 5 years after starting the medications. Depression and anxiety were also noted. Brain MRIs showed no structural change and the MMSE score was 28 out of 30 while she was 36 years old. A genetic analysis with a targeted NGS panel identified a homozygous c.991G > T (p.D331Y) mutation in *PLA2G6*. The clinical details were described previously [5]. This study was approved by the Institutional Review Board of National Taiwan University Hospital. Written informed consent was obtained from the participants.

### Systematic review of previous reports of patients with PLAN

We searched the PubMed database for all English literature that had the terms: “PLA2G6 mutation” or “phospholipase A2 group VI gene mutation”. We selected case reports or case series of patients with genetically confirmed PLAN, published in 2008–2019. Patients with childhood onset were excluded. We selected patients with adult-onset PLAN (onset age older than 18 years) and affected family members within the same family. However, family members with onset age less than 18 years were excluded. We summarized the clinical, genetic, and imaging characteristics of different genotypes to elucidate adult-onset *PLA2G6*.

We identified 32 patients from 23 articles that described patients with adult-onset PLAN. We did not enroll patients with onset age less than 18 years old. Including our three patients, we analyzed data for 35 patients. The clinical characteristics and genetic findings are summarized in Table 1 [1–3, 6–32]. The mean age of onset was 26.3 ± 8.6 years. The mean age of examination was 30.4 ± 5.5 years. At the initial presentation, 10 patients (28.6%) had neuropsychiatric symptoms, rather than motor dysfunction. These symptoms included cognitive decline (*n* = 2) and depression with or without anxiety (*n* = 8) (Table 1). The motor phenotypes were heterogeneous. Fourteen patients (40.0%) had dystonia-parkinsonism, 17 patients (48.6%) had levodopa-responsive, early-onset parkinsonism (EOPD), 3 patients (8.6%) had hereditary spastic hemiparesis (HSP), and 1 patient (2.9%) had cerebellar ataxia without signs of parkinsonism. The age at onset and gender distribution in patients with different clinical sub-groups were comparable (Table 2). The percentage of combined dystonia was higher in the group with dystonia-parkinsonism than in other groups (*P* < 0.001; Table 2). Of the 35 patients during examination, the main neurological signs included parkinsonism in 24 patients (68.6%), neuropsychiatric symptoms in 19 patients (54.3%), pyramidal signs with increased deep tendon reflexes in 19 patients (54.3%), dystonia in 12 patients (34.3%) and ataxia in 6 patient s (17.1%) (Table 1). Brain MRIs were performed in all patients. Structural abnormalities were observed in 26 patients (74.3%), including cortical atrophy (*n* = 18, 51.4% of all 35 patients), cerebellar atrophy (*n* = 5, 14.3% of all 35 patients), and hypointensity or suspected iron accumulations in the globus pallidus (observed in T2-weighted MRIs; *n* = 3, 8.6% of all 35 patients). The percentage of cerebellar atrophy detected on brain MRIs was lower in patients with EOPD than in those with dystonia-parkinsonism, HSP, or ataxia (*P* = 0.03, Table 2). Tc-99 m TRODAT SPECT scans were performed in 12 patients, and all showed impaired and asymmetrical dopamine transporter activity. Six patients underwent brain PET studies, and all revealed hypometabolism in the cortical areas.

**Table 1:** Clinical, genetic, and imaging characteristics of patients with genetically confirmed adult-onset PLAN in the literature

Author, year	Patient	Ethnicity	Genotype	Type	Sex	AAO	Initial symptoms	Main symptoms during examination					MRI
								EPS	Dystonia	Pyramidal signs	Ataxia	Psychiatric	
**Homozygous p.D331Y mutations (N = 5)**													
Shi et al. 2011 [6]	P1	Chinese	Homo c.991G > T (p. D331Y)	EOPD	M	37	Gait disturbance	+	–	–	–	–	nl
Xie et al. 2015 [7]	PA	Chinese	Homo c.991G > T (p. D331Y)	EOPD	M	36	Gait disturbance	+	–	–	–	–	nl
	PB	Chinese	Homo c.991G > T (p. D331Y)	EOPD	M	36	Right hand tremor	+	–	–	–	–	nl
This study	P2	Chinese	Homo c.991G > T (p. D331Y)	EOPD	F	30	Slowing gait	+	–	–	–	D, P, A	nl
	P3	Chinese	Homo c.991G > T (p. D331Y)	EOPD	F	26	Right leg clumsiness	+	–	+	–	D, A	nl
**Compound heterozygous p.D331Y/ other mutations (N = 5)**													
Lu et al. 2012 [8]	P3	Chinese	Compound hetero c.991G > T (p. D331Y)/c.1077G > A (M358IfsX)	DP	F	19	Unsteadiness and bradykinesia	+	+	–	+	P,C	CoA, CeA
Chen et al. 2018 [9]	P3	Chinese	Compound hetero c.991G > T(p.D331Y)/c.1982C > T	DP	M	29	Walking difficulty	+	–	–	+	–	CeA
	P4	Chinese	Compound hetero c.991G > T(p.D331Y)/ c.2218G > A (p.G740R)	HSP	F	31	Gait disturbance	–	–	+	+	–	CeA
Ji et al. 2019 [10]	P1	Chinese	Compound hetero c.991G > T (D331Y)/c.1648delC	Ataxia	F	30	Imbalance	–	–	+	+	D	CeA
This study	P1	Chinese	Compound hetero c.991G > T (p. D331Y)/c.1077G > A (M358IfsX)	DP	M	18	Depression and psychosis	+	+	–	–	D, P, C	CoA, CeA
**Homozygous Mutations other than p.D331Y (*N*** **= 17)**													
Paisa’n-Ruiz et al., 2008 [3]	P1 of F1	Indian	Homo c.2222G > A (p.R741Q)	DP	F	26	Cognitive decline	+	+	+	–	D, C	CoA
	P1 of F2	Pakistani	Homo c.2239C > T (p.R747W)	DP	F	18	Foot drag	+	+	+	–	C	CoA
Sina et al. 2009 [11]	P1	Iranian	Homo c.C1894T (p.R632W)	DP	M	25	Foot drag	+	+	+	–	C	CoA
	P2	Iranian	Homo c.C1894T (p.R632W)	DP	M	22	Foot drag	+	+	+	–	C	CoA
	P3	Iranian	Homo c.C1894T (p.R632W)	DP	F	21	Foot drag	+	+	+	+	C	CoA
Agarwal et al., 2012 [12]	P1	Scandinavian	Homo c.G238A (p.A80T)	EOPD	F	22	Depression	+	+	+	–	D, A, C	I
Virmani et al., 2014 [13]	P1	Indian	Homo c.2222G > A (p.R741Q)	DP	F	25	Depression and psychosis	+	+	+	–	D, P	CoA
	P2	Indian	Homo c.2222G > A (p.R741Q)	DP	F	22	Depression	+	+	+	–	–	CoA
Malaguti et al. 2015 [14]	P	Italian	Homo c.C1547T (p.A516W)	DP	F	27	Stiff leg sensation	+	+	+	–	C	I
Giri et al. 2016 [15]	P	Turkish	Homo c.2239C > T (p.R747W)	EOPD	F	27	Left limb slowness	+	–	–	–	D,P,C	CoA
Ozes et al. 2017 [16]	P2	Turkish	Homo c.2239C > T (p.R747W)	HSP	M	21	Scissoring gait	–	–	+	+	–	I
Koh et al. 2018 [17]	D II-3	Japanese	Homo c.1904G > A (p.R635Q)	HSP	F	66	Gait impairment	+	–	+	–	–	nl
Bohlega et al. 2018 [18]	P1 of F1	Saudi Arabian	Homo c.2222G > A (p.R741Q)	EOPD	F	26	Depression, bradykinesia	+	–	+	–	D, C	CoA
	P2 of F1	Saudi Arabian	Homo c.2222G > A (p.R741Q)	EOPD	M	22	Depression, tremor	+	–	+	–	D, C	CoA
	P3 of F1	Saudi Arabian	Homo c.2222G > A (p.R741Q)	EOPD	F	23	Bradykinesia	+	–	+	–	C	CoA
	P1 of F2	Saudi Arabian	Homo c.2222G > A (p.R741Q)	EOPD	M	25	Cognitive decline	+	–	+	–	C	CoA
Rohani et al. 2018 [19]	P	Saudi Arabian	Homo p. Ala681Cysfs*92	DP	M	18	Bradykinesia, tremor	+	+	+	–	P	nl
**Compound Heterozygous mutations other than p.D331Y (N = 8)**													
Yoshino et al. 2010 [20]	PA	Japanese	Compound hetero c.C216A (p. p.F72L) /c.G1904A (p.R635Q)	EOPD	F	20	Resting tremor, unsteady gait	+	–	–	–	D,P,C	CoA, I
	PB1	Japanese	Compound hetero c.C1354T (p.Q452X) /c.G1904A (p.R635Q)	EOPD	M	25	Bradykinesia, gait disturbance	+	–	–	–	C	CoA
	PB2	Japanese	Compound hetero c.C1354T (p.Q452X) /c.G1904A (p.R635Q)	EOPD	M	30	Bradykinesia, gait disturbance	+	–	–	–	P,C	CoA
Bower et al., 2011 [21]	P1	European	Compound hetero c.C4A (p.Q2K)/Del Ex 3 (pL71_S142del)	DP	F	18	Depression	+	+	+	–	D	GP
Kim et al. 2015 [22]	P1	Korean	Compound hetero c.G1039A (p.G347R) /c.C1670T (p.S557L)	DP	F	22	Unsteady gait and falls	+	+	+	+	C	CeA, I
Wirth et al. 2017 [23]	P1	Caucasian	Compound hetero c.109C > T (p.R37X)/ c.2321G > T (p.Ser774Iso)	EOPD	M	23	Depression and anxiety	+	+	–	–	D,P,A	nl
	P2	Caucasian	Compound hetero c.758G > T (Gly253Val)/c.2341G > A (Ala781Thr)	EOPD	M	27	Left leg tremor and anxiety	+	–	+	–	P,A,C	nl
Chen et al.2018 [9]	P2	Chinese	Compound heteroc.668C > T (p.P223L) /c.1915G > A(p.A639T)	EOPD	M	27	Walking difficulty	+	–	+	–	–	CeA, GP, I

Abbreviations: +, present; −, absent or not reported; *F1* family 1; *P1* patient 1; *DP* dystonia-parkinsonism; *EOPD* early-onset Parkinson’s disease; *HSP* hereditary spastic hemiplegia; *AAO* age at onset; *EPS* extrapyramidal sign; *MRI* magnetic resonance imaging; *D* depression; *P* psychosis; *A* anxiety; *C* cognitive decline; *CoA* Cortical atrophy; *CeA* Cerebellar atrophy; *GP* T2- hypointensity in globus pallidus; *I* Iron accumulation; *nl* normal; *hypo* hypometabolism; *NA* not available

**Table 2:** Comparison of patients with different clinical subtypes of PLAN

Characteristics	Dystonia-Parkinsonism *N* = 14	Early-onset PD *N* = 17	HSP *N* = 3	Ataxia *N* = 1	*P*-value
Age at onset, years	21.7 ± 3.9	27.2 ± 5.1	46.0 ± 23.5	30	0.10
Age at examination, years	32.5 ± 4.9	33.7 ± 8.1	N.A.	31	0.26
Sex, male	5 (35.7%)	10 (58.8%)	1 (33.3%)	0	0.38
Main symptoms and signs					
Parkinsonism	14 (100.0%)	17 (100.0%)	0	0	1.00
Dystonia	13 (92.8%)	2 (11.8%)	0	0	< 0.001**
Pyramidal sign	11 (78.6%)	8 (47.1%)	3 (100%)	1	0.32
Cognitive decline	9 (64.3%)	10 (58.8%)	1 (33.3%)	0	0.91
Depression/Anxiety	4 (28.6%)	7 (41.2%)	1 (33.3%)	1	0.78
Psychosis	4 (28.6%)	6 (35.3%)	0	0	0.93
Brain MRI findings					
Cortical atrophy	9 (64.3%)	8 (47.1%)	0	0	
Cerebellar atrophy	3 (21.4%)	1 (5.9%)	1 (33.3%)	1	0.03*
Hypo-intensity in GP	1 (7.1%)	1 (5.9%)	0	0	0.98

Data are the number (%) or the mean ± SD. PLAN, Phospholipase A2 group VI-associated neurodegeneration; PD, Parkinson’s disease; HSP, hereditary spastic paraparesis; MRI, magnetic resonance imaging; GP, globus pallidus. **P < 0.05; **P < 0.01. *P*-values that compare individual characteristics between groups with dystonia-parkinsonism and early-onset PD were evaluated with an analysis of variance. Variables without a normal distribution were compared with the Kruskal-Wallis test, the non-parametric equivalent of the independent sample t-test

### Comparison of patients with different PLAN-related genotypes

We next assigned patients to one of four groups, according to genotype, to reappraise potential genotype-phenotype correlations (Table 3). All three of our patients had either homozygous or compound heterozygous p.D331Y mutations. Therefore, we compared the following four genotype groups: homozygous p.D331Y mutations (*n* = 5); compound heterozygous p.D331Y mutations with other variants (n = 5); homozygous mutations other than p.D331Y (*n* = 17); and compound heterozygous mutations that did not include p.D331Y (*n* = 8) (Table 3). Of note, we found that the p.D331Y mutation in either the homozygous or compound heterozygous state, was more prevalent among Chinese and East Asians than among other ethnic groups (*P* < 0.001 for homozygous p.D331Y mutations; *P* = 0.04 for compound heterozygous p.D331Y with other variants, Table 3). Specifically, all patients with homozygous p.D331Y mutations and more than 85% of patients with compound heterozygous p.D331Y mutations were of Chinese ethnicity, which suggested a potential common founder effect. On the other hand, homozygous mutations other than p.D331Y were more prevalent among South Asians and Middle Eastern individuals, including Pakistanis, Saudi Arabians, Iranians, and Turks (*P* < 0.001, Table 3).

**Table 3:** Comparison of patients with different PLAN-related genotypes

Characteristics	Homozygous p.D331Y mutations N = 5	Compound heterozygous p.D331Y/ other mutations N = 5	Homozygous Mutations other than p.D331Y N = 17	Compound Heterozygous mutations other than p.D331Y N = 8	*P*-value
Age at onset, years	33.0 ± 4.8	25.4 ± 6.3	23.2 ± 11.0	16.8 ± 9.9	0.23
Sex, male	2 (50.0%)	2 (40.0%)	6 (35.3%)	5 (62.5%)	0.41
Ethnicity					
Chinese	5 (100.0%)	5 (100.0%)	0	1 (12.5%)	< 0.001**
East Asian	0	0	1 (4.8%)	4 (50.0%)	0.01*
South Asian	0	0	4 (19.0%)	0	0.16
Middle Eastern	0	0	14 (66.7%)	0	< 0.01**
Caucasian	0	0	2 (9.5%)	3 (37.5%)	0.11
Main clinical subtypes					
Dystonia-parkinsonism	0	3 (60.0%)	9 (52.9%)	2 (25.0%)	0.07
Early-onset PD	5 (100.0%)	0	6 (35.3%)	6 (75.0%)	0.01*
HSP	0	1 (20.0%)	2 (11.8%)	0	0.78
Ataxia	0	1 (20.0%)	0	0	0.68

Data are the number (%) or the mean ± SD. PLAN, Phospholipase A2 group VI-associated neurodegeneration; PD, Parkinson’s disease; HSP, hereditary spastic paraparesis. **P < 0.05; **P < 0.01. *P*-values that compare individual characteristics between four groups with different genotypes were evaluated with an analysis of variance. Variables without a normal distribution were compared with the Kruskal-Wallis test, the non-parametric equivalent of the independent sample t-test

Phenotypically, all patients with homozygous p.D331Y mutations (*n* = 5) had EOPD, with a later age of onset (mean age of onset 33.0 ± 4.8 years), compared to patients with other genotypes (*P* = 0.03, Table 3). All five of these patients initially had an asymmetric onset of parkinsonism symptoms and responded well to levodopa. However, three of these five patients reported levodopa-induced dyskinesia. None exhibited dystonia, ataxia, an oculomotor abnormality, or autonomic dysfunction. Of note, the MRI findings of all five patients with homozygous p.D331Y showed no obvious structural changes, including T2-hypointensity in the globus pallidus. On the other hand, patients with mutations other than homozygous p.D331Y had an earlier age of onset (*P* = 0.03, Table 3) and often presented with dystonia-parkinsonism, rather than early-onset levodopa-responsive parkinsonism (30.7–57.1%, Table 3). All carriers of mutations other than homozygous p.D331Y had abnormal brain MRI findings, including cortical and cerebellar atrophy, but without T2-hypointensity in the globus pallidus or iron accumulation.

Among 34 patients having mutations other than p.D331Y, nine patients carried homozygous c.2222G > A (p.R741Q) mutations and four patients had homozygous c.2239C > T (p.R747W) mutations. Of those 9 patients having homozygous p.R741Q mutations, six (66.7%) had neuropsychiatric symptoms as the initial presentation and five (55.6%) had features of dystonia-parkinsonism. Furthermore, of those 4 patients with homozygous p.R747W mutations, two had HSP phenotypes without signs of parkinsonism, one had dystonia-parkinsonism, and another one was clinically diagnosed with levodopa-responsive EOPD.

## Discussion and conclusions

We described the clinical, genetic, and neuroimaging aspects of three patients with adult-onset PLAN. One patient had compound heterozygous p.D331Y and M358IfsX frame-shift mutations and presented with dystonia-parkinsonism. The other two patients had homozygous p.D331Y mutations and presented with levodopa-responsive early-onset parkinsonism*.* After identifying more than 20 *PLA2G6* mutations in previous reports [1–3, 6–32], we observed that the c.991G > T (p. D331Y) mutation in *PLA2G6* was almost exclusively found in Chinese patients, suggesting a common founder effect of this variant in Chinese populations. Moreover, patients with homozygous c.991G > T (p.D331Y) mutations showed purely early-onset parkinsonism features with good levodopa responses. In contrast, patients with other genotypes, including compound heterozygous c.991G > T (p.D331Y) mutations and other variants, predominantly presented with dystonia-parkinsonism and HSP. Our results further support previous studies that *PLA2G6* mutations had both clinical and genetic heterogeneity. Moreover, our findings suggested that the c.991G > T (p.D331Y) mutation was a potentially common founder mutation in populations of Chinese ethnicity.

The homozygous c.991G > T (p.D331Y) mutation in *PLA2G6* was first identified in patients diagnosed with levodopa-responsive EOPD (25, 30). Consistent with initial reports, our two patients with this same mutation had comparable phenotypes and there were no signs of iron accumulation in the globus pallidus in brain MRIs. The *PLA2G6* gene encodes a group of VIA calcium-independent phospholipase A2 (iPLA2β) enzymes, which participate in various cellular functions, including phospholipid metabolism, membrane homeostasis, calcium signaling, apoptosis, and mitochondrial function. Thus, these functions could be perturbed by *PLA2G6* gene mutations [24]. A homozygous c.991G > T (p.D331Y) knock-in mouse model exhibited dopaminergic neuron degeneration in the substantia nigra, caused by mitochondrial dysfunction, elevated endoplasmic reticulum stress, and transcriptional abnormalities, conforming its pathogenicity in neuronal degeneration [24]. A previous in vitro study showed that patients with the homozygous c.991G > T (p.D331Y) mutation had 30% enzymatic activity remaining in the PLA2G6 protein by using the modified kit originally designed for cytosolic Ca2^+^-dependent PLA2 (cPLA2) (cPLA2 Assay Kit, Cayman Chemicals) in HEK293 T cells transfected with human mutant constructs (25). An incomplete loss of the iPLA2β enzyme could partially explain the relatively milder clinical and neuroimaging phenotypes of patients with homozygous c.991G > T (p.D331Y) mutations, compared to patients with other mutations in *PLA2G6*.

The crystal structure of iPLA2β is complex, and the different mutation sites are disparately located on the enzyme [1]. Different mutation sites in the different domains of iPLA2β can lead to different changes in enzymatic activities; however, iPLA2β enzyme activity is the crucial factor that affects the clinical phenotypes of PLAN [33]. For example, the activity of iPLA2β was reduced by 70%, with some remaining enzyme activity, in cells that expressed p.D331Y, compared to wild-type cells [6]; in contrast, iPLA2β with the p.H597fx69 frame-shift mutation exhibited less than 6% enzyme activity compared to the wild-type iPLA2β [34]. The compound heterozygous genotype of p.D331Y and p.M358IfsX mutations was first reported in a Chinese patient that presented with dystonia-parkinsonism [8]. Our first index patient with these same mutations shared similar clinical manifestations, including early age at onset, prominent dystonia, depression, and cognitive decline. The MRI scans in both patients did not show signs of iron accumulations in globus pallidus. The c.1077G > A (p.M358IfsX) mutation, which is located at the splicing junction, resulted in c.1074_1077del.GTCG, and caused aberrant RNA splicing, which resulted in a frame-shift mutation. We hypothesized that this frame-shift mutation might perturb iPLA2β enzyme activity. Indeed, patients with homozygous p.D331Y mutations often presented with levodopa-responsive EOPD, but patients with compound heterozygous p.D331Y and p.M358IfsX mutations often presented with dystonia-parkinsonism, with features of dystonia, pyramidal signs, ataxia, and psychiatric comorbidities. Accordingly, we speculated that iPLA2β enzyme activity might be less in neurons that expressed the p.M358IfsX mutation than in neurons that expressed the p.D331Y mutation. Further functional studies are needed to clarify the molecular mechanism that leads to the clinical heterogeneity associated with different mutations in *PLA2G6*.

In addition to levodopa-responsive EOPD and dystonia-parkinsonism, 3 patients (from the literature review) having *PLA2G6* mutations and displayed HSP, which findings extended our current understanding of the spectrum of clinical phenotypes associated with adult-onset PLAN. Moreover, one patient had compound heterozygous c.991G > T (D331Y) and c.1648delC mutations and presented with prominent ataxia, pyramidal signs, and depression, but did not show signs of parkinsonism or dystonia [10]. That presentation was also rare in adult-onset PLAN. In addition to diverse motor symptoms, neuropsychiatric symptoms comprise one of the main features of adult-onset PLAN. Our index patient with compound heterozygous p.D331Y and p.M358IfsX mutations had severe depression, and one of the two patients with a homozygous p.D331Y mutation also had depression and prominent psychotic symptoms. Many reports also found neuropsychiatric symptoms or behavioral changes in the initial presentations of patients with *PLA2G6* mutations [3, 12, 13, 18, 23, 25, 27]. Early cognitive decline or psychiatric symptoms with motor symptoms, such as parkinsonism or dystonia, should prompt clinicians to include PLAN in their differential diagnoses. In those cases, genetic testing for *PLA2G6* mutations are warranted, even when brain MRIs show no iron accumulation in the globus pallidus. Moreover, due to the susceptibility to developing psychotic symptoms, dopaminergic agents for treating parkinsonism symptoms in these patients should be carefully titrated and closely monitored to identify psychiatric complications during treatment.

This study had some limitations. First, due to the rarity of the disease, the number of cases was limited. This limitation might have attenuated the statistical power of the results in the comparison of clinical phenotypes among different *PLA2G6* mutations. Moreover, due to the diverse clinical manifestations, including motor and neuropsychiatric involvements, the clinical assessments might not have been comprehensive, in every case, in the literature review. Furthermore, although our study focused on adult-onset PLAN, some family members of the index patient may have symptoms since the childhood and we still enrolled these patients into the analysis to have a better understanding of the clinical presentation of the same mutation within the family. Future studies should include thorough clinical, functional imaging, and genetic analyses, with a long-term follow-up, to provide a better understanding of the correlations between genotype and phenotype in PLAN.

In summary, our findings suggested that adult-onset PLAN could present with purely early-onset levodopa-responsive parkinsonism features, with no brain iron accumulation, particularly in patients with homozygous c.991G > T (p.D331Y) mutations. The homozygous c.991G > T (p.D331Y) mutations were exclusively found in patients of Chinese ethnicity, suggesting a common founder effect. Patients with compound heterozygous mutations had heterogeneous phenotypes, including dystonia-parkinsonism, HSP, and ataxia, and these were often combined with diverse neuropsychiatric symptoms. The wide intra- and inter-familial phenotypic variability of adult-onset PLAN may contribute from other environmental and/or genetic modifiers that might probably modulate the disease presentation. Future studies encompassing whole genome sequencing in patients with PLAN are needed to delineate the potential modifier genes of this disease.

## Supplementary information

**Additional file 1 Supplementary Table 1.** The 40 candidate genes involved in PD and related neurodegenerative disorders that were used for targeted NGS in the study.

## Data Availability

All the relevant raw data in the current study will be freely available to any scientist wishing to use them for non-commercial purposes, without breaching participant confidentiality.

## Abbreviations

PLA2G6: Phospholipase A2 group VI
PLAN: Phospholipase A2 group VI (*PLA2G6*) mutations associated with neurodegeneration
