# Supplementary material for: Genotype-phenotype correlations of adult-onset PLA2G6-associated Neurodegeneration: case series and literature review
Source: BMC Neurol. 2020 Mar 17;20:101. doi: 10.1186/s12883-020-01684-6 (PMC7076921; doi:10.1186/s12883-020-01684-6)
Supplement: Supplementary file 1 — Additional file 1 Supplementary Table 1. The 40 candidate genes involved in PD and related neurodegenerative disorders that were used for targeted NGS in the study. [file 12883_2020_1684_MOESM1_ESM.docx]

Supplementary Table 1. The 40 candidate genes involved in PD and related neurodegenerative disorders that were used for targeted NGS in the study.

| **Gene** | **OMIM number** | **Chromosome location** | |
| --- | --- | --- | --- |
| *SNCA* | 163890 | 4q21 | chr4: 90,635,250–90,769,466 |
| *Parkin* | 602544 | 6q26 | chr6: 161,768,590–163,148,834 |
| *UCHL1* | 191342 | 4p13 | chr4: 41,258,898–41,270,446 |
| *PINK1* | 608309 | 1p36.12 | chr1: 20,959,948–20,978,004 |
| *DJ-1* | 602533 | 1p36.23 | chr1: 8,021,714–8,045,342 |
| *LRRK2* | 609007 | 12q12 | chr12: 40,618,813–40,763,086 |
| *ATP13A2* | 610513 | 1p36.13 | chr1: 17,312,453–17,338,467 |
| *GIGYF2* | 612003 | 2q37.1 | chr2: 233,562,015–233,725,289 |
| *HtrA2* | 606441 | 2p13.1 | chr2: 74,756,532–74,760,683 |
| *PLA2G6* | 603604 | 22q13.1 | chr22: 38,111,495–38,192,100 |
| *FBXO7* | 605648 | 22q12.3 | chr22: 32,870,707–32,894,818 |
| *VPS35* | 601501 | 16q11.2 | chr16: 46,693,589–46,723,144 |
| *SYNJ1* | 604297 | 21q22.1 | chr21: 34,001,069–34,100,351 |
| *DNAJC13* | 614334 | 3q22.1 | chr3: 132,136,553–132,257,876 |
| *DNAJC6* | 608375 | 1p31.3 | chr1:65,775,218-65,881,552 |
| *CHCHD2* | 616244 | 7p11.2 | chr7:56,169,266-56,174,187 |
| *GBA* | 606463 | 1q21 | chr1:155,204,239-155,214,653 |
| *SMPD1* | 607608 | 11p15.4 | chr11:6,411,644-6,416,228 |
| *NPC1* | 607623 | 18q11.2 | chr18:21,111,463-21,166,581 |
| *NPC2* | 601015 | 14q24.3 | chr14:74,946,643-74,960,084 |
| *TAF1* | 313650 | Xq13.1 | chrX:70,586,114-70,685,855 |
| *GCH1* | 600225 | 14q22.1-q22.2 | chr14:55,308,724-55,369,542 |
| *ATP1A3* | 182350 | 19q13.2 | chr19:42,470,734-42,498,428 |
| *PANK2* | 606157 | 20p13 | chr20:3,869,742-3,904,502 |
| *COQ2* | 609825 | 4q21.23 | chr4: 84,184,977–84,206,067 |
| *APP* | 104760 | 21q21.3 | chr21: 27,252,861–27,543,138 |
| *PSEN1* | 104311 | 14q24.2 | chr14: 73,603,143–73,690,399 |
| *PSEN2* | 600759 | 1q42.13 | chr1: 227,058,273–227,083,804 |
| *MAPT* | 157140 | 17q21.31 | chr17: 43,971,701–44,105,699 |
| *GRN* | 138945 | 17q21.31 | chr17: 42,422,454–42,430,474 |
| *CHMP2B* | 609512 | 3p11.2 | chr3:87,276,413-87,304,698 |
| *TYROBP* | 604142 | 19q13.12 | chr19:36,395,303-36,399,211 |
| *TREM2* | 605086 | 6p21.2 | chr6:41,126,244-41,130,924 |
| *PRNP* | 176640 | 20p13 | chr20:4,666,797-4,682,234 |
| *Rab39B* | 300774 | Xq28 | chrX:154,487,526-154,493,852 |
| *SOD1* | 147450 | 21q22.11 | chr21: 33,031,935–33,041,243 |
| *FUS* | 137070 | 16p11.2 | chr16: 31,191,431–31,206,192 |
| *TARDBP* | 605078 | 1p36.22 | chr1: 11,072,679–11,085,549 |
| *OPTN* | 602432 | 10p13 | chr10: 13,142,082–13,180,276 |
| *VCP* | 601023 | 9p13.3 | chr9: 35,056,065–35,072,739 |

PD, Parkinson’s disease; NGS, next generation sequencing
